# Development of a Low-Cost System for 3D Orchard Mapping Integrating UGV and LiDAR

**DOI:** 10.3390/plants10122804

**Published:** 2021-12-17

**Authors:** Harold F. Murcia, Sebastian Tilaguy, Sofiane Ouazaa

**Affiliations:** 1 Facultad de Ingeniería, Universidad de Ibagué, Ibagué 730003, Colombia; sebastian.tilaguy@unibague.edu.co; 2 Centro de Investigación Nataima, Corporación Colombiana de Investigación Agropecuaria-AGROSAVIA, Km 9 vía Espinal-Chicoral, Espinal 733529, Colombia; souazaa@agrosavia.co

**Keywords:** sensing, unmanned ground vehicle, 3D reconstruction, point clouds, crop monitoring

## Abstract

Growing evaluation in the early stages of crop development can be critical to eventual yield. Point clouds have been used for this purpose in tasks such as detection, characterization, phenotyping, and prediction on different crops with terrestrial mapping platforms based on laser scanning. 3D model generation requires the use of specialized measurement equipment, which limits access to this technology because of their complex and high cost, both hardware elements and data processing software. An unmanned 3D reconstruction mapping system of orchards or small crops has been developed to support the determination of morphological indices, allowing the individual calculation of the height and radius of the canopy of the trees to monitor plant growth. This paper presents the details on each development stage of a low-cost mapping system which integrates an Unmanned Ground Vehicle UGV and a 2D LiDAR to generate 3D point clouds. The sensing system for the data collection was developed from the design in mechanical, electronic, control, and software layers. The validation test was carried out on a citrus crop section by a comparison of distance and canopy height values obtained from our generated point cloud concerning the reference values obtained with a photogrammetry method. A 3D crop map was generated to provide a graphical view of the density of tree canopies in different sections which led to the determination of individual plant characteristics using a Python-assisted tool. Field evaluation results showed plant individual tree height and crown diameter with a root mean square error of around 30.8 and 45.7 cm between point cloud data and reference values.

## 1. Introduction

Information on geometrical and structural characteristics of crops reveals important insight and provides decisive knowledge for management within an orchard. Numerous techniques and sensors have been used to extract useful information efficiently and effectively and thus facilitate early detection and action tasks for farmers. These technologies are expected to revolutionize agriculture, enabling timely decision-making, promising significant cost reduction, and increasing the crop yield [1]. Such decisions allow the effective application of farm inputs, supporting the pillars of precision agriculture: apply the right practice, at the right place, at the right time, and with the right quantity. Within this wide range of sensing possibilities, Light Detection and Ranging (LiDAR) technologies are one of the options of interest given their ability to generate 3D LiDAR point clouds such as elevations models for altitude and slope mapping. The processing of 3D LiDAR point clouds also enhances crop analysis by building semantic models with qualitative information at different dimensions, which can be abstracted with machine learning algorithms. The highest-end LiDARs have an accurate and fast response for three-dimensional measurements. Moreover, they can provide additional information with respect to the conventional, e.g., multiple echoes response, the associated returned energy, and the pulse width time of each return [2]. These top devices are more expensive, bigger, and heavier for use with mobile vehicles, which makes access and application difficult. Mobile crop mapping consists of digital modeling of the crop with a sufficiently dense point cloud to describe the morphological characteristics of the terrain and the plants. This information can be used to assess the growth of the crop, estimate geometric tree characteristics, detect fruits, characterize a crop, estimate a crop yield, monitor the crop canopy, determine the crop biomass, leaf area index, and other high-throughput phenotyping parameters [3]. In this context, a vehicle navigates in the crop environment and accumulates the point clouds obtained by the LiDAR taking into account its location relative to an initial position. In this way, the successive accumulation of the transformed point clouds to a reference pose results in reconstruction in the form of a point cloud with at least three dimensions of information. Low-cost possibilities are part of the prospects and future development of such technologies according to [3]. The challenge with low-cost systems involves the deployment of simple devices for 3D reconstruction, e.g., the absence of an accurate device with good resolution for the measurement of the position of the mobile on the trajectory and the use of two-dimensional LiDAR sensors, which offer only one plane of information.

Most of the reported work uses a Terrestrial Laser Scanner (TLS) configuration, which can be considered as a fixed scanner with a large sensing range over a tripod with at least one rotational actuator. Recent research has focused on applications such as Stem–Leaf segmentation [4,5], wheat height detection [6], canopy characterization [7,8], corn detection [9], leaf area evaluation [10], leaf index estimation [11], biomass estimation [12], and plant structure phenotyping for an indoor environment with fixed structure implementations but using a transnational movement [13]. A second configuration defined in this paper—Mobile Terrestrial Laser Scanner (MTLS)—consists of a similar TLS configuration but includes a mobile platform with a location sensor such as a Global Position System (GPS) [14] or a computational process to estimate it. Moreover, there are other mobile platforms where a LiDAR device is mounted on-board of an unmanned vehicle (UGV or UAV) with a navigation system and computational algorithms in a continuous operation mode to locate and map the environment at the same time. This concept has been addressed in the field of robotics for many years under the concept of Simultaneous Localization and Mapping (SLAM) [15]. Consulted applications use the generated point clouds for fruit detection, yield prediction [14], canopy characterization [16], automatic panicles detection [17], high-throughput phenotyping [5,18], low cost phenotyping [16], and fast phenotyping [19], among others. Table 1 summarizes the applications, crops, and used sensors on recent works.

The development of a high-resolution phenotyping platform integrated with postprocessing technology offers the opportunity to assess complex traits more effectively. Compared to other platform types, UGVs reduce personnel time to cover large test plots and provide more accurate measurement because they can operate at constant speed and avoid human error. They can also be less constrained by operating times or load capacity.

The quality of the point cloud obtained depends on all the system elements, however the LiDAR sensor and localization subsystem have a great impact on the 3D mapping. Popular LiDAR sensors used on these applications are 3D Velodyne VLP-16, 3D Velodyne HDL64, 2D Sick LMS-400, and Faro Focus X330, which provides approximately hundreds of thousand points/second and have low sensitivity to optical noise in outdoor conditions. Likewise, the use of positioning devices, including sensor fusion and Real-Time Kinematics (RTK) techniques, which provide real-time location corrections, implies a large increase in the quality of 3D models. Although the development and evaluation of sensor-based technologies for estimating plant indices is an area of active development and current scanning platforms encompass both airborne and ground-based systems, there are still costs associated with the chosen devices, data processing software, and data integration that may limit the widespread adoption of these systems.

The present work focuses on terrestrial applications. In this paper, we describe the development and assessment of a low-cost mobile scanner based on a 2D LiDAR sensor on-board a ground robot to navigate semi-controlled through an orchard. It is shown how low-cost devices can allow the mapping and generation of point clouds for the purpose of incorporating remote sensing techniques in crop management applications.

## 2. Materials and Methods

### 2.1. Terrestrial Mobile Robot

**Figure 1:**
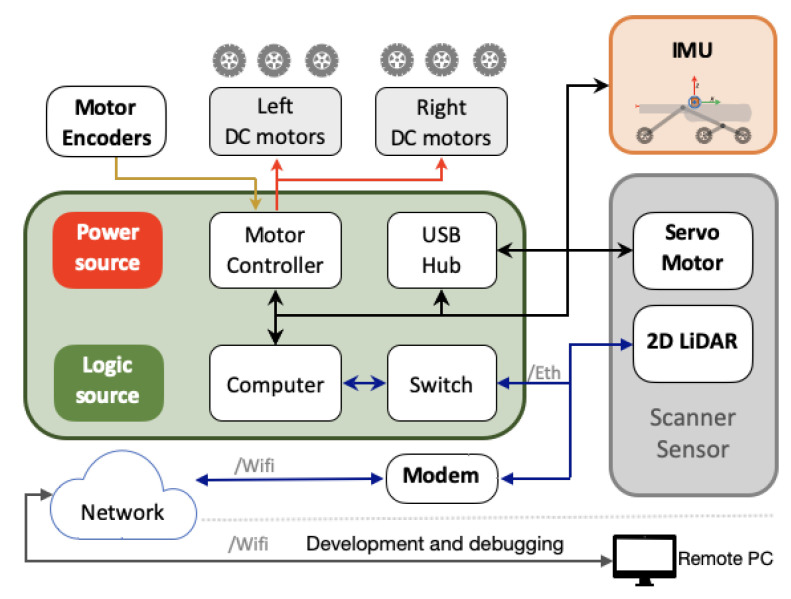
Functional diagram of the 3D mapping system.

The used platform in this work is an own development to make 3D models of orchards and small crops with low-cost devices in comparison with commercial versions. The operating parameters were defined from problem requirements to select the needed components, then the prototype was designed in mechanical, electronic, and software layers. The design and implementation details of the platform called alphaRover are presented in Section 2.1.1 and Section 2.1.2. This Unmanned Ground Vehicle (UGV) has a skid-steer configuration with six wheels and rocker-bogie suspension. The robot uses Pololu gearmotors with magnetic encoders of 6533 PPM and a Xsens Mti-30 sensor to estimate the robot position and trajectory, which is a is a full gyro-enhanced Attitude and Heading Reference System (AHRS) with roll/pitch (static|dynamic) 0.2${}^{\circ }$|0.5${}^{\circ }$ and yaw 1.0${}^{\circ }$. A NVIDIA Jetson TK1 board with Ubuntu 14.04 and Robotic Operating System ROS indigo manipulates the motors energy per each side (left and right) by using a Roboclaw 2 × 15A unit. The robot was operated by using a wifi network which connected a remote pilot computer with a gamepad Logitech F710. The on-board scanner subsystem includes a servomotor Dynamixel AX-12A which supports a LiDAR with a tilt inclination of 45${}^{\circ }$ with respect to the ground. Figure 1 illustrates the functional scheme of the system and its main elements. At the end of the route, the system records the data in a raw file, which is processed in subsequent stages to generate the point cloud and to determine the crop parameters that are derived from the point cloud.

#### 2.1.1. Hardware Design

Mechanical design consisted in the selection of shapes, sizes, and materials for the construction of the robot structure, which was digitally designed by using SolidWorks software to evaluate the skid-steering architecture and a rocker-bogie suspension [21]. These configurations were defined aiming to minimize the effect of the irregular terrain profile in experimental conditions, reducing strong disturbances on the perception devices. The design of the six-wheel bogie suspension system has a shock absorber located in the rocker arm to protect the motors at the rear of the robot by reducing the force of impacts when overcoming obstacles. This improvement increases the reliability of the structure on rough or uneven terrain by allowing each of the wheels is in contact with the surface at all times. The mechanical design of rocker-bogie begins with considerations of the work ground. Therefore, we consider obstacles less than 5 cm high and 7 cm long. Using Equation (1), the minimum necessary radius of the wheel is 7.4 cm. Therefore, we select wheels with a radius of 7.5 cm.

(1) $$r_{min}=\frac{h_{obs}^{2}+w_{obs}^{2}}{2h_{obs}}$$

**Figure 2:**
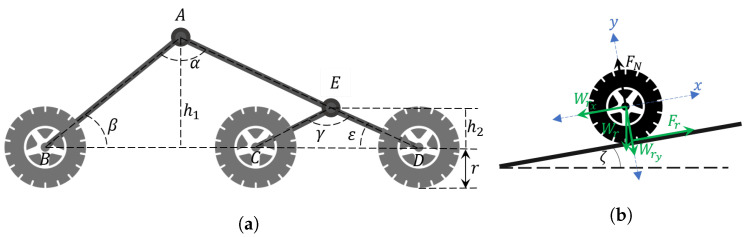
Design of the rocker-bogie suspension system: (**a**) complete rocker-bogie suspension; (**b**) wheel force diagram.

Taking into account the rocker-bogie scheme shown in Figure 2a, the suspension design begins by defining the vehicle size and the top angle of the fixed part α. Furthermore, to simplify and make the design systematic, we assume the angle β as $\frac{pi-\alpha }{2}$ rad, so therefore $\overset{↔}{AB}$ is determined as a hypotenuse. The $\overset{↔}{BD}$ segment and $h_{1}$ distance are in function of the vehicle and wheels sizes. Thus, the segment $\overset{↔}{AD}$ can be obtained from Equation (2). By assuming $h_{2}$ distance as the wheel diameter $\overset{↔}{DE}$ and $\overset{↔}{CE}$ can be determined as as hypotenuses in function of the ε and γ angles, respectively. Thus, $\overset{↔}{AE}$ is calculated as the difference between $\overset{↔}{AD}$ and $\overset{↔}{ED}$.

(2) $${\overset{↔}{AD}}^{2}={\overset{↔}{BD}}^{2}+{\overset{↔}{AB}}^{2}-2\left(\overset{↔}{AB}\right)\left(\overset{↔}{BD}\right)\cos\beta$$

In motor selection, it is important to consider requirements when working in the worst case. We assume a surface with an inclination angle of $\zeta_{s}$, an estimated mass of the vehicle $m_{r}$, an estimated load capacity $m_{L}$, a minimum linear speed $v_{min}$, a minimum linear acceleration $a_{min}$, the coefficient of friction μ, and a safety factor SF to oversize the design. The process began by analyzing the Figure 2b, where all forces are concentrated on one wheel to simplify the design. As each wheel has its own motor and the motors are the same (with the same reference), the obtained torque was defined as the sum of the torque contributed by each motor. Finally, the design parameters obtained are presented in Table 2.

The coefficient of friction was obtained from a table from the materials literature. The total mass that the motors have to move was defined as $m_{T}=m_{r}+m_{L}$. Therefore, the components of the robot weight vector were calculated as

(3) $$\begin{array}{cccc} \; & W_{r_{y}} & = & -m_{T}\cos\left(\zeta_{s}\right)g\\ \; & W_{r_{x}} & = & -m_{T}\sin\left(\zeta_{s}\right)g \end{array}$$

The normal force has the same magnitude but the opposite direction that *y* component of weight, $F_{N}=-W_{r_{y}}$. The friction force was defined as $F_{r}=-\mu F_{N}$, where the negative sign represents the opposition of movement. The total torque in the wheel was defined as

(4) $$\sum \tau =I\dot{\omega }$$

where $\dot{\omega }$ is the angular acceleration and *I* is the moment of inertia, which, considering the wheel as a solid cylinder, then $I=\frac{1}{2}mr^{2}$. Assuming there is no external force on the wheel, and using the parameters from Table 2, the total torque for the alphaRover is

(5) $$\tau =rF_{r}+\frac{1}{2}mra=6.9\;N.m$$

Then, given the number of wheels, the torque for each motor $\tau_{m}$ must be

(6) $$\tau_{m}=\frac{\tau }{6}SF=1.955\;N.m$$

After a review of possibilities, the selected motor was the Pololu 37Dx73L gearmotor, an 8 watt motor with a 2.2 N.m torque at 12 VDC. Finally, the validation design was made by comparing the linear speed $v_{m}$ of the alphaRover using the selected motor in relation with the desired speed from Table 2. In this way, the velocity in RPM must be converted to $\frac{m}{s}$:

(7) $$v_{m}=RPM\frac{2\pi }{60}r=0.7854\;\frac{m}{s}$$

As $v_{m}>v_{min}$, the selected engine fulfills the design requirements.

On the one hand, the power consumption of the control elements and sensors was considered in order to define the necessary power supply for these electronic components and the appropriate control device. In addition, all design considerations include a desired minimum operating time, an autonomy time considered as $t_{o}\approx 1\;h$. The design starts with an analysis of required energy, the consumption of control elements and sensors, which is presented in the Table 3.

The elements are connected with DC/DC converters which have an average efficiency of 80%. Therefore, the total power consumption is approximately 51.25W. Then, considering a LiPo battery of 6 cells, the average current consumption is calculated as

(8) $$I_{b}=\frac{P_{T}}{N_{C}*V_{C}}$$

where $P_{T}$, $N_{C}$, and $V_{C}$ are the total energy consumption, the number of cells, and the voltage per cell, respectively. Then, taking into account $I_{b}$ current, the operating time $t_{o}$, and a safety factor to oversize the design, the minimum capacity of the battery to guarantee the desired operating time is

(9) $$C_{Logic_{min}}=I_{b}t_{o}SF\approx 4\;\mathrm{Ah}$$

For the power analysis in motors, the mass of the real vehicle was measured m≈14kg. This mass was used to determinate the average working torque with a constant vehicle movement speed:

(10) $${\bar{\tau }}_{M}=\frac{rmg}{6}\sin\left(\frac{\pi }{2}\right)\approx 1.1\;N.m$$

For the scanning application, it is important to move at a constant and slow speed to ensure good resolution. These design considerations are explained in Section 2.1.2. The angular speed is determined from the reference linear speed of the robot:

(11) $$\omega_{sacan}=\frac{v_{sacan}}{r}$$

Thus, with the new torque ${\bar{\tau }}_{M}$ and the motor speed $\omega_{sacan}$, the power consumption of each motor is

(12) $$P_{M}=\tau \omega_{sacan}$$

For the nominal motor voltage a 3 cell battery was selected. To guarantee the same current, each motor on the same side was connected on the same channel. Therefore, the current per channel is

(13) $$I_{ch}=3\frac{P_{M}}{V}$$

The driver current was selected considering the worst case and an efficiency of $\eta_{driver}=0.8$:

(14) $$I_{stall}=\frac{8\;W}{V}$$

(15) $$I_{stall_{max}}=3I_{stall}SF$$

The power controller for motors must support at least 1.6A per channel. The battery selection must take into account the consumption of the motors, the efficiency of the selected controller, and the desired operating time:

(16) $$C_{Power_{min}}=2\frac{I_{ch}}{\eta_{driver}}t_{o}SF\approx 6.3\;Ah$$

Finally, the battery capacity selected was of 6Ah.

#### 2.1.2. Software Design

The software is composed by two layers: preprocessing and processing. The preprocessing layer is directly related to the fusion of the acquired data from the different perception elements involved in the generation of the three-dimensional point cloud, which represents the environment mapped by the robot. The processing layer determines the crop parameters from the point-cloud generated and user support. The robot uses an Ubuntu 14.04 operating system and Robotic Operating System ROS in its Indigo version. The system allows a manual or semi-controlled operation mode defined by the pilot from an external computer connected to the robot’s network via WiFi. In the manual mode, the energy applied to the motors depends exclusively on the joystick’s game-pad signal; conversely, in the semi-controlled mode, it has an intermediate wheel speed control layer that assists the action on the motors, facilitating the navigation even on slopes or irregular terrains. This last mode is explained in detail in the Section 2.1.3.

**Figure 3:**
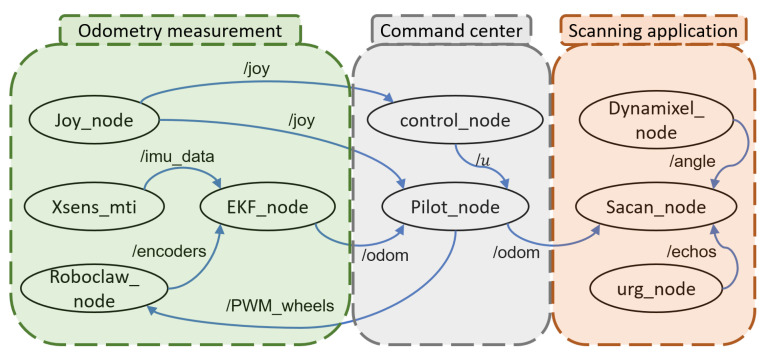
Computational software diagram in ROS nodes representation, from left to right the first, second, and third group of nodes. The nodes communicate with each other with messages, which are organized into specific categories called topics.

Figure 3 illustrates the computational software diagram of the system represented in ROS nodes (circles) and ROS topics (connection lines) which were developed or reconditioned from ROS programming community in C++ and Python languages. The first group of nodes corresponds with direct measurements and actions such as remote game-pad signals (Joy), Inertial Measurement Unit IMU (Xsens), Extended Kalman Filter (EKF) and motor control unit (Roboclaw). The second group of nodes contents the administration nodes which perform tasks such as remote connection with the pilot (Pilot), perception devices management, action elements management, data registration, and the synchronized data recording to generate the offline point cloud at the end of the robot’s paths (Control). The last group contents the nodes from the scan unit, composed by a servomotor (Dynamixel), the 2D LiDAR sensor (URG), and the central subsystem (Scan) to control both elements.

#### 2.1.3. Embedded Cruise Control

The second manipulation mode presents some operation advantages for the pilot such as making movements at constant speed in front of perturbations in the wheels due to ground irregularities, upward or downward slopes, and minor obstacles; in this way, it is possible to make soft displacements avoiding accelerations that can affect the instruments and the robot. Additionally, it is possible to maintain a working rhythm independently of small discharges in the battery, as the control regulates the energy applied on the motors according to perturbations or voltage changes. To ensure a constant speed of the mobile during the scanning process, it was necessary to implement a speed control on each set of wheels, with a great robustness to cope with different terrains and obstacles, in addition to the effects of vibration and mechanical degradation. This design is based on the consideration of a second-order dynamic system:

(17) $$G\left(s\right)=k\frac{\omega_{o}^{2}}{s^{2}+2\zeta \omega_{o}s+\omega_{o}^{2}}$$

A PID controller with robustness greater than 80% was designed using FRtool [22] with a mathematical model presented in the Equation equation and its estimated parameters. The settling time $\left(t_{s}\right)$ is another important parameter for the controller design, which has a trade-off with robustness. As our application does not require large accelerations, the speed does not have to change rapidly, so its settling time could be as slow as $t_{s}\approx 2$ seconds. Finally, the maximum overshoot parameter $\left(M_{p}\right)$, which is always desired to be as small as possible, was defined with a tolerance of less than 5%. Table 4 presents the estimated model parameters for the Equation (17), the design parameters for the control, and the obtained constants for the PID controller in its discrete implementation described in the following equation:

(18) $$C\left(z\right)=K_{p}+K_{i}\frac{T_{s}}{z-1}+\frac{K_{d}}{T_{s}}\left(z-1\right)$$

**Figure 4:**
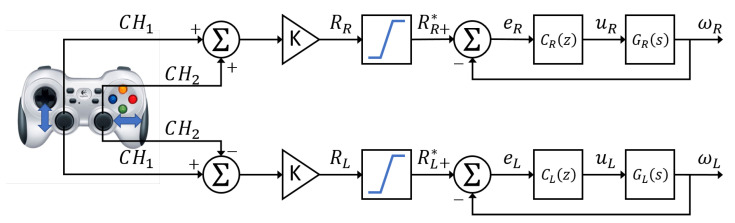
Control scheme for the speed control system. $CH_{1}$ and $CH_{2}$ represent the gamepad gestures; $\omega_{R}$ and $\omega_{L}$ represent the controlled wheel speed for the right side and the left side, respectively.

Figure 4 illustrates the control scheme, which was implemented as difference equations into the “control node” rosnode as shown in Figure 3. The gestures of Channels 1 and 2 are generated in the gamepad to generate the acceleration and direction signal respectively. A gain *K* transforms the operated signals from the gamepad in the $\left[\frac{\mathrm{rad}}{\mathrm{seg}}\right]$ control set-points. These reference signals are saturated to guarantee the values of the angular speed limits. C(s) and G(s) are transfer function blocks that represent the PID controllers and the motors, respectively. The importance of speed control lies in giving the robot the ability to follow a constant speed reference and give it a certain degree of settlement in the presence of disturbances either from the ground or from its own construction characteristics.

### 2.2. Pose Estimation

**Figure 5:**
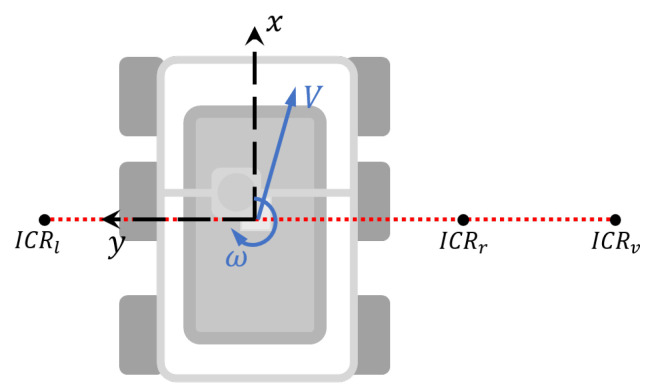
ICR on the vehicle plane.

The skid-steer topology is based on Instantaneous Centers of Rotation ICR parameters. The ${ICR}_{v}$ is the point where the vehicle’s rotation takes place without translation movement. The ${ICR}_{r}$ and ${ICR}_{l}$ are the traction points representation on the local frame for the right and left sets of wheel, respectively [23]. Figure 5 shows our local reference framework and the position of each ICR point, which are necessary to describe the movement of the vehicle.

The behavior of the position of a six-wheel skid-steer topology robot was modeled using equations presented in [24]. The kinematics relation between wheel velocities ($v_{r_{i}}=r\omega_{i}$) and the vehicle’s translational velocity *v* are expressed as

(19) $$\left[\begin{array}{c} v_{x}\\ v_{y}\\ \omega \end{array}\right]=\frac{r}{y_{{ICR}_{r}}-y_{{ICR}_{l}}}\left[\begin{array}{cc} -y_{{ICR}_{l}}\alpha_{r} & y_{{ICR}_{r}}\alpha_{l}\\ x_{{ICR}_{v}}\alpha_{r} & -x_{{ICR}_{v}}\alpha_{l}\\ \alpha_{r} & -\alpha_{l} \end{array}\right]\left[\begin{array}{c} \omega_{r}\\ \omega_{l} \end{array}\right]$$

where $\alpha_{r,l}$ are correction factors, ω is the angular speed around *z* axis, and magnitude of the vehicle’s translational speed can be described as $\left|v\right|=\sqrt{v_{x}^{2}+v_{y}^{2}}$. To simplify the analysis, in this document $\alpha_{c}=\alpha_{l}=\alpha_{r}=1$ is considered; also is assumed $x_{{ICR}_{v}}=0$, given the center of mass is in the center of vehicle. Finally, given each set of wheels is symmetric, is assumed that $y_{{ICR}_{r}}=-y_{{ICR}_{l}}$. In this way, the model showed in (19) is simplified as

(20) $$\left[\begin{array}{c} v\\ \omega \end{array}\right]=\frac{r\alpha_{c}}{2y_{ICR}}\left[\begin{array}{cc} -y_{ICR} & y_{ICR}\\ 1 & -1 \end{array}\right]\left[\begin{array}{c} \omega_{r}\\ \omega_{l} \end{array}\right]$$

ICR coordinates are variable parameters which can be estimated from experimental measurements [25]. This estimation is possible thanks to the cruise control implementation, where $\left(v_{r},v_{l}\right)$ are regulated. Equation (21) shows the relationship ${y_{ICR}}_{\left(v_{r},v_{l},\phi_{mag}\right)}$.

(21) $$y_{ICR}\approx \frac{\int v_{r}dt-\int v_{l}dt}{2\Delta \theta_{mag}}$$

where $\Delta \theta_{mag}$ is the change of robot orientation angle, which for this work is the measurement from a magnetometer in order to calculate the $y_{ICR}$. Some related works simplify the analysis in the form of a differential model; however, it is important to assess the need of extending the classical differential model. In this way, the related work in [25] introduces an expression to quantify the efficiency of the differential model with respect to the presented skid-steer model. This expression compares the relationship between *L* and the difference of ICR points of each side as follows:

(22) $$\chi =\frac{L}{y_{{ICR}_{r}}-y_{{ICR}_{l}}},\;\;\left(0\leq \chi \leq 1\right)$$

When the χ value is 1 it implies that the differential model is enough to describe the movement of vehicle. However, based on some experiments, our χ value reached a maximum of 0.03, which indicates that even the best case is far from being explained by a differential model. Once our kinematic model is defined, we proceed with the discrete implementation. Therefore, it was necessary to rewrite the model in term of differential equations (Δ), where $V=\frac{\Delta S_{p}}{T_{s}}$ and $\omega =\frac{\Delta \theta }{T_{s}}$,

(23) $$\left[\begin{array}{c} \Delta S_{p}\\ \Delta \theta \end{array}\right]=\frac{r\alpha_{c}T_{s}}{2y_{ICR}}\left[\begin{array}{cc} -y_{ICR} & y_{ICR}\\ 1 & -1 \end{array}\right]\left[\begin{array}{c} \omega_{r}\\ \omega_{l} \end{array}\right]$$

where $\Delta S_{p}$ and Δθ is the magnitude of the change in position and orientation, respectively. $T_{s}$ is the sampling period defined as 100ms. Considering the new representation and defining that $\Delta S_{p_{k}}=S_{p_{k}}-S_{p_{k-1}}$ and $\Delta \theta_{k}=\theta_{k}-\theta_{k-1}$. Equation (19) can be rewritten as

(24) $$\left[\begin{array}{c} x_{k}\\ y_{k}\\ \theta_{k} \end{array}\right]=\left[\begin{array}{c} x_{k-1}\\ y_{k-1}\\ \theta_{k-1} \end{array}\right]+\left[\begin{array}{c} \Delta S_{p_{k-1}}\cos\left(\theta_{k-1}+\frac{\Delta \theta_{k}}{2}\right)\\ \Delta S_{p_{k-1}}\sin\left(\theta_{k-1}+\frac{\Delta \theta_{k}}{2}\right)\\ \Delta \theta_{k} \end{array}\right]$$

Equation (24) corresponds to a nonlinear model of estimated position, where $x_{k}$ and $y_{k}$ correspond to the position of the vehicle at xaxis and yaxis, respectively, and $\theta_{k}$ is the orientation angle in the inertial robot frame, which is obtained from an IMU. To correctly estimate the pose and orientation it was necessary to implement an Extended Kalman Filter (EKF); this algorithm is necessary to mix the information of sensors and minimize the measurement noise. Then, we initialize $R_{k}$, $Q_{k}$, and *P*, where $Q_{k}$ is the covariance matrix of the process noise, $R_{k}$ is the covariance matrix of the measurement noise, and *P* the covariance error matrix, which must initialize with very large values.

**Figure 6:**
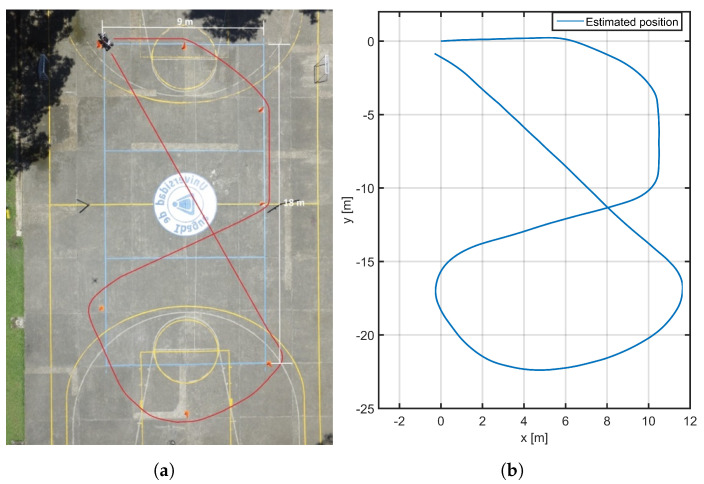
Result of trajectory estimation: (**a**) test setup and robot trajectory; (**b**) XY trajectory estimation.

The algorithm was implemented in a rosnode programmed in Python language mentioned into the Section 2.1.2. A pose estimation algorithm for the vehicle was implemented in a real-time node and was tested in a sports field using a trajectory defined by traffic cones an monitored from an aerial video, as shown in Figure 6a. This trajectory was intended to verify the behavior of the EKF implemented in trajectories with turns in all directions. The implemented EKF only estimated the *yaw* angle and the *x* and *y* coordinates. The *z* coordinate and tilt angles (*roll* and *pitch*) are assumed to be 0, in consideration of flat terrain thanks to the absorption of obstacles by the rocker-boogie topology and because of the simplified implementation of the mapping algorithm.

Figure 6b shows the trajectory reconstruction obtained in a rosbag file where EKF data were captured from the test carried out on the sports field. This result not only presents the correct implementation of the kinematic model, but also minimizes the measurement noise from the encoders and the IMU.

### 2.3. Lidar Mapping

**Figure 7:**
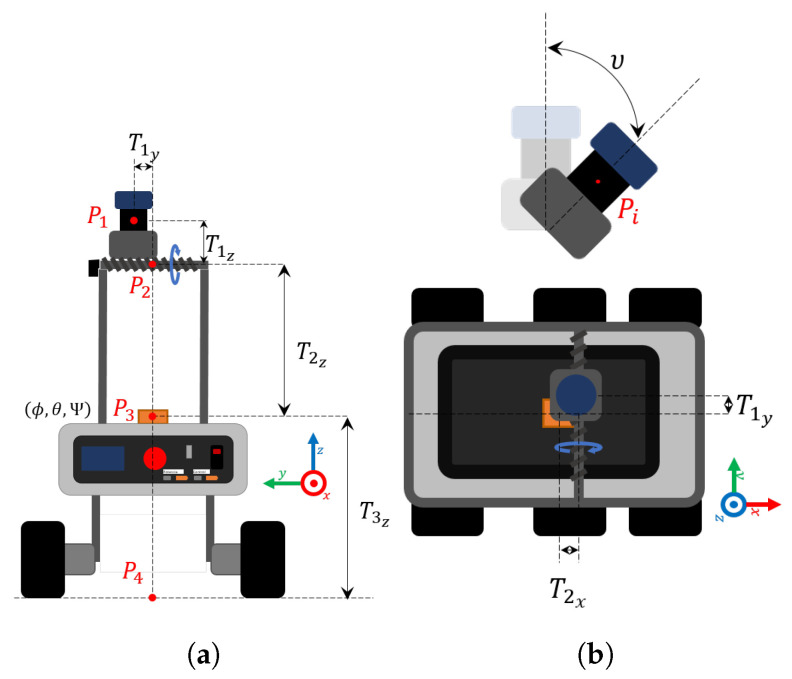
Schematic diagram of the mapping system and notations for different coordinated frame points to transform initial points from LiDAR to the reference frame $P_{o}$: (**a**) rear view of vehicle, (**b**) top view of the vehicle.

The preprocessing involved the reconstruction of the navigation environment and each of the trees from the LiDAR data with the scanning laser rangefinder 2D Hokuyo UTM-30LX-EW, which is a small device for outdoor robotic applications with a millimeter resolution with a 30 m range and 270${}^{\circ }$ field of view. The angular resolution is 0.25${}^{\circ }$ and the accuracy of the distance measurement is greater than 3 cm, depending on distance, the angle of incidence of the light beam and illumination of the environment. The simplest way to create the 3D model of the crop with a 2D laser sensor is by scanning the scene while the sensor remains static with a fixed inclination. The purpose of our mapping system is to acquire the LiDAR responses in a local coordinate frame and convert it to an absolute reference coordinate frame centered in $P_{o}$ point, which corresponds with the the initial point of the robot’s mission. According to a previous work [26], the final 3D point cloud in $P_{o}$ consists in a XYZ space in function of LiDAR horizontal angles *H* and radial distances *R*, as well as the servomotor vertical angle υ, the XY position derived from the rover trajectory and some system parameters which represent the distances and possible angular deviations. With these measurements, the 3D position of an illuminated point *P* is given by a sequence of transformations from the 2D point $P_{i_{\left(R,H\right)}}$ to $P_{1}$, $P_{1}$ to $P_{2}$, $P_{2}$ to $P_{3}$, and $P_{3}$ to $P_{o}$, respectively, as shown in Figure 7. In a general representation, these transformations are formulated from a basic transformation matrix $T_{p}$ with [4 × 4] size:

(25) $$T_{p}=R_{z}\cdot R_{y}\cdot R_{x}\cdot T$$

where *T* is a three-dimensional translation transformation with three parameters: $t_{x}$, $t_{y}$, and $t_{z}$. $Rx_{p}$ represents a rotation transformation around *X* axis (pitch), $Ry_{p}$ represents a rotation transformation around *Y* axis (roll), and $Rz_{p}$ represents a rotation transformation around *Z* axis (yaw), for a total of six parameters per frame transformation: three angles (φ, ϑ, Ψ) and three distances ($t_{x}$, $t_{y}$, $t_{z}$). Each one of the above-mentioned matrices are described as follows:

(26) $$T=\left[\begin{array}{ccccccc} 1 & \; & 0 & \; & 0 & \; & t_{x}\\ 0 & \; & 1 & \; & 0 & \; & t_{y}\\ 0 & \; & 0 & \; & 1 & \; & t_{z}\\ 0 & \; & 0 & \; & 0 & \; & 1 \end{array}\right]$$

(27) $$R_{x}=\left[\begin{array}{cccc} 1 & 0 & 0 & 0\\ 0 & c(\varphi ) & -s(\varphi ) & 0\\ 0 & s(\varphi ) & c(\varphi ) & 0\\ 0 & 0 & 0 & 1 \end{array}\right]$$

(28) $$R_{y}=\left[\begin{array}{cccc} c(\vartheta ) & 0 & s(\vartheta ) & 0\\ 0 & 1 & 0 & 0\\ -s(\vartheta ) & 0 & c(\vartheta ) & 0\\ 0 & 0 & 0 & 1 \end{array}\right]$$

(29) $$R_{z}=\left[\begin{array}{cccc} c(\Psi ) & -s(\Psi ) & 0 & 0\\ s(\Psi ) & c(\Psi ) & 0 & 0\\ 0 & 0 & 1 & 0\\ 0 & 0 & 0 & 1 \end{array}\right]$$

According to the above, the final XYZ reconstruction is composed of four transformations from $P_{i}$ to $P_{o}$, which are based on the Equation (25). Each coordinate of $P_{i}$ is determined from the measured distance from the LiDAR to the illuminated object and the horizontal angle at which the beam was generated. Let us assume *R* as a diagonal matrix containing the range values in meters obtained from the LiDAR sensor for each horizontal angle beam.

(30) $$R=\left[\begin{array}{ccc} R_{1} & \; & \\ \; & \ddots & \\ \; & \; & R_{N} \end{array}\right]$$

*H* comprises the horizontal angles of the 2D LiDAR for each range *R* measurement and is considered as a matrix of size [N×1], where *N* is the number of beams or samples per each 2D LiDAR scan. In our case, the sensor is symmetrical and has a measuring range of 240${}^{\circ }$, therefore $H_{0}$ has a value of −120${}^{\circ }$ and $\Delta_{H}={\left(240/N\right)}^{\circ }$.

(31) $$H=H_{0}+\left[\begin{array}{c} 0\\ \Delta_{H}\\ \vdots \\ N\Delta_{H} \end{array}\right]$$

The corresponding equation for the matrix $P_{1}$ with size [4×N], which is obtained from initial data is given as

(32) $$P_{1}=\left[\begin{array}{c} X_{1}\\ Y_{1}\\ \vec{0}\\ \vec{1} \end{array}\right]=\left[\begin{array}{c} R\cdot sin(H)\\ R\cdot cos(H)\\ 0\cdots 0\\ 1\cdots 1 \end{array}\right]$$

The transformation matrix from $P_{1}$ to $P_{2}$ is given as

(33) $$Tp_{1}=Ry_{\left(\upsilon \right)}\cdot T_{1}$$

where $Ry_{\left(\upsilon \right)}$ is the rotation matrix according to the motor angle υ. The transformation matrix from the servo motor axis $P_{2}$ to the centered IMU frame $P_{3}$ is given as

(34) $$Tp_{2}=Rz_{\left(\Psi \right)}\cdot Ry_{\left(\theta \right)}\cdot Rx_{\left(\phi \right)}\cdot T_{2}$$

In which the movement pitch is represented by θ, the movement roll is represented by ϕ, and the movement yaw is represented by Ψ. A third transformation consists of a vertical translation to the reference *Z* level at $P_{4}$, by including the distance from IMU sensor to the floor.

(35) $$Tp_{3}=T_{3}$$

The last transformation matrix contents the rover movement information. The matrices $T_{\left(Xk\right)}$ and $T_{\left(Yk\right)}$ include the estimated motion information of the robot on its *X* and *Y* axes in the absolute frame with respect to the $P_{o}$ point by using an EKF based on the work in [27].

(36) $$Tp_{4}=T_{\left(Yk\right)}\cdot T_{\left(Xk\right)}\cdot T_{4}$$

With the following assumptions: (1) a null error on angle around *Z*-axis: $e_{\Psi }=\Psi_{real}-\Psi_{estimated}=0$; (2) a minimal effect given angular deviation errors on physical implementation: $Rx_{1}=Rz_{1}=Rx_{3}=Ry_{3}=Rz_{3}=Rx_{4}=Ry_{4}=Rz_{4}\approx 0$; (3) a correct XY UGV pose estimation $e_{xy}=P_{real}-P_{estimated}=0$; (4) $P_{o}$ is the position $P_{\left(x,y,z,\theta ,\phi ,\Psi \right)}$ of the UGV at the zero moment; and (5) a flat navigation environment without elevations on the ground, finally, Equation (37) summarizes the complete transformation:

(37) $$P_{o}=Tp_{4}\cdot Tp_{3}\cdot Tp_{2}\cdot Tp_{1}\cdot P_{1}$$

In which $P_{1}$ corresponds with the first frame obtained from the initial LiDAR data, $Tp_{1}$, $Tp_{2}$, $Tp_{3}$, and $Tp_{4}$ are the transformation matrices to transform the data to the reference frame centered in $P_{o}$.

### 2.4. Field Data Collection

**Figure 8:**
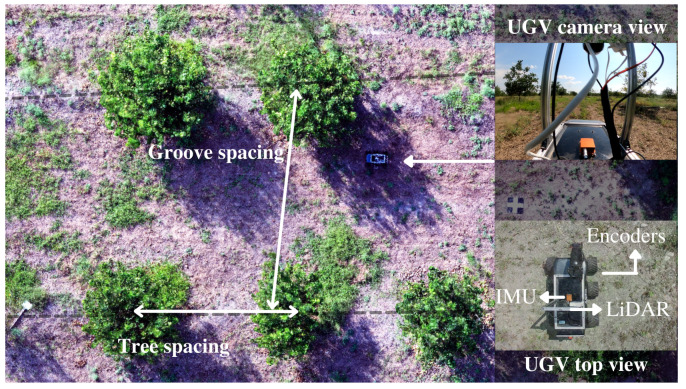
The mobile 3D LiDAR mapping system into the citrus crop.

The system was evaluated on 40 citrus trees distributed along a groove of the crop, and for the reconstruction of the 3D model, from the total group, a region consisting of 24 plants was selected to validate the determination of morphological parameters in each of the plants. The orchard site is located at Agrosavia Nataima Research Center, Chicoral, Tolima, Colombia (Latitude: 4°21′21.54″ N, Longitude: 54°55′40.59″ W, Altitude: 409 m above sea level). The tree to tree spacing in the row was approximately 5.0 m with 8.0 m between furrows and approximately average height of 2.5 m. Figure 8 illustrates the experimental field and the used platform among two planting lines into the crop.

Ground truth sampling was performed from a 3D reference point cloud using a DJI Mavic 2 pro aerial vehicle and Pix4D professional mapping software with its mapping and modeling tool mapping and the data capture application capture [28]. Once the point cloud was obtained, a point cloud processing tool with a user interface called CloudCompare was used to obtain measurements of average canopy height and radius for each tree in two of the crop rows [29].

During the field data collection, the UGV moved through a crop furrow in an on-the-fly mode, with continuous rosbag data recording process. The rosbag collects system variables in a synchronized manner: IMU data have a sampling rate of 100 Hz, the signals coming from the vehicle’s encoders have a sampling rate of 55 Hz, and the LiDAR responses, as well as other robot variables such as current, voltage, temperature, and communication information. The data collection experiment of 115 m furrow took approximately three minutes. The sampling rate of the LiDAR is 40 Hz, each scan contains approximately 1440 light beams per return, i.e., approximately 7200 scans and 10 million points in the case of the first echoes. However, given the geometry of the system, not all of the 360${}^{\circ }$ range is exploited, so the number of measurements per scan is reduced to less than half, and although the sensor reports three echoes in range and intensity, not all points contain information. Once the vehicle has finished its route, the rosbag recording process was stopped and the raw data were extracted for offline processing on a different computer than the robot.

### 2.5. Crop Parameters Estimation

The 3D reconstructed trees were then used to extract the tree to tree distances and furrow to furrow distance for a section of two furrows, as well as canopy heights and crown diameter through a developed algorithm in Python. The height of the tree canopy was defined as the height from the ground to the height where the highest leaves are found, in other words the distance in *z* axis from a $z_{ref}$ point to the highest point of the tree. The algorithm starts with the determination of the reference plane to determine canopy height $z_{ref}$ by using least squares method and some picked points from the ground and the center point $Pt_{(i,j)\left(x,y\right)}$ of each tree (i) per furrow (j) from the point cloud top view, in order to calculate the tree to tree distances, the furrow to furrow average distance and the canopy heights for each tree. The $z_{max}$ parameter is obtained by finding the maximum value in a cylindrical region $R_{(i,j)\left(t\right)}$ (see Equation (38)) with a user-defined radius $r_{(i,j)\left(t\right)}$ centered on point $P_{(i,j)\left(x,y\right)}$, which is the tree center defined from the graphical representation.

(38) $$R_{(i,j)\left(t\right)}:\forall \left\{x,y,z\right\}|\left(\sqrt{{\Delta x}^{2}+{\Delta y}^{2}}\leq r_{(i,j)\left(t\right)}\right)\wedge \left(z\geq z_{ref}\right)$$

where $\Delta x=x-x_{o(i,j)}$ and $\Delta y=y-y_{o(i,j)}$, and $x_{o}$ and $y_{o}$ are the center coordinates of the tree obtained from $Pt_{i,j}$. Then, once the $z_{ref}$ and center points are defined for each tree, the method obtains the parameter as follows: let be $Pt_{(i,j)\left(x,y\right)}$ a $\left[N_{t}\times 2N_{F}\right]$ matrix with the xy coordinates of the centers of each tree, $Ht_{\left(j\right)}$ is a $\left[N_{t}\times N_{F}\right]$ matrix with the high of each tree, $T_{dst\left(j\right)}$ is a $\left[N_{t}-1\times N_{F}\right]$ matrix with the distances tree to tree per furrow and $F_{dst}$ a $\left[N_{F}-1\times 1\right]$ matrix with the distances furrow to furrow:

(39) $$Ht\left[i,j\right]=\max\left(R_{(i,j)\left(z\right)}\right)-z_{ref}$$

with $i=0,1,\ldots ,N_{t}$ and $j=0,1,\ldots ,N_{F}$.

(40) $$T_{dst}\left[i,j\right]=\sqrt{{\left(x_{o(i,j)}-x_{o(i-1,j)}\right)}^{2}+{\left(y_{o(i,j)}-y_{o(i-1,j)}\right)}^{2}}$$

with $i=1,2,\ldots ,N_{t}$ and $j=0,1,\ldots ,N_{F}$.

To find the furrow to furrow distance, it is not possible to guarantee the same number of trees per furrow or the perfect perpendicularity to each other; therefore, $S_{F}$ is a first degree polynomial generated from minimizing the squared error of all the Pt per furrow defined as $S_{F}\left(x_{a}\right)=ax_{a}+b$, where $x_{a}$ is the vector of *x*-axis values in the plot, and the proposed method consist to measure the average distance between lines.

(41) $$\begin{array}{c} F_{dst_{0}}\left[i\right]={\left[\sqrt{{\left(S_{F}\left(x_{a(0,j)}\right)-S_{F}\left(x_{a(0,j-1)}\right)\right)}^{2}}\right]}^{T}\\ F_{dst_{f}}\left[i\right]={\left[\sqrt{{\left(S_{F}\left(x_{a(f,j)}\right)-S_{F}\left(x_{a(f,j-1)}\right)\right)}^{2}}\right]}^{T}\\ F_{dst}=\frac{F_{dst_{0}}-F_{dst_{f}}}{2} \end{array}$$

with $i=0,1,\ldots ,N_{t}$ and $j=1,2,\ldots ,N_{F}$.

The method details are shown in Algorithm 1. The algorithm takes as inputs the generated point cloud from the crop, and the user-defined parameters in the assisted interface.
**Algorithm 1:** Crop parameters determination
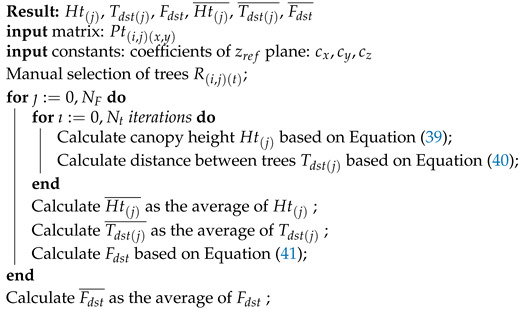


## 3. Results

### 3.1. Generated Point Clouds

**Figure 9:**
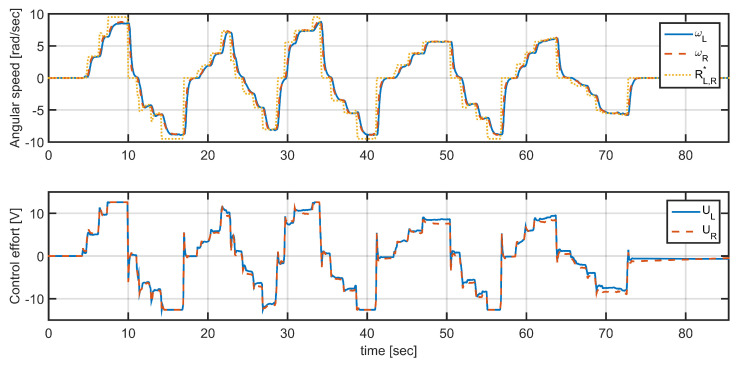
Speed setting of one of the left motors (blue) and one of the right motors (red) with respect to the angular speed reference (yellow). The horizontal axis is in samples, with a sample time $T_{s}=0.1$ s.

To validate the design presented in the Section 2.1.3, the vehicle was lifted off the ground to avoid external disturbance and to see the closed-loop response of angular velocity and its ability to follow a reference imposed by gamepad in the same conditions of the experiment for the model parameter estimation. The results of this experiment are presented in Figure 9.

**Figure 10:**
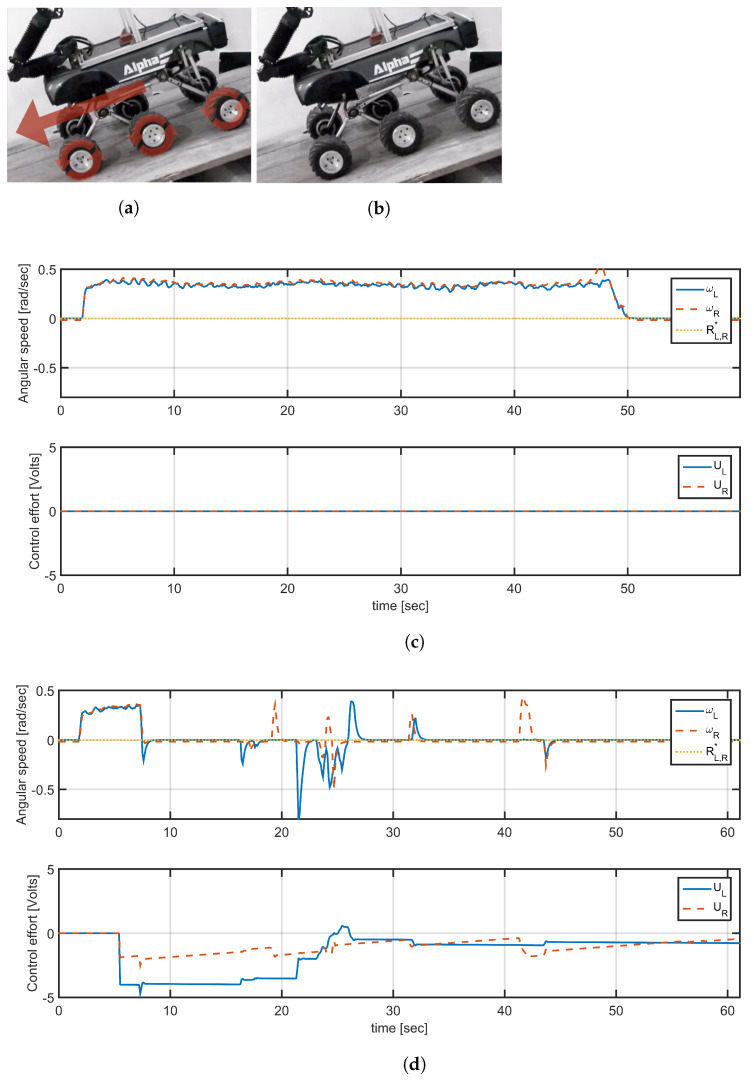
Speed control response in rad/s to a 30${}^{\circ }$ incline: (**a**) the robot rolls on the ramp, (**b**) angular speed responses without control, (**c**) the robot holds its position without rolling effect, and (**d**) angular speed responses with. Left motors speed (blue), sped of one of the right motors speed (red), angular speed reference (yellow).The horizontal axis is in samples, with a sample time $T_{s}=0.1$ s.

To evaluate the scope of the controller a new experiment was carried out to compare vehicle responses with and without speed control. The vehicle was placed on a flat platform with an approximate inclination of 30deg, where in open-loop condition the vehicle slides down as its weight exceeds the inertia of motors, as shown in Figure 10a. Figure 10c shows speed reference (yellow line) located at $0\;\frac{\mathrm{rad}}{s}$, the orange and blue curves correspond to the measured angular velocity of the motors right and left, respectively, which show an angular velocity different from zero in both cases. A new test was performed under the same circumstances, but this time with the speed control. Figure 10d shows the angular speed of the right and left wheels when implementing the wheel speed control for the same reference value. In this case, although there are some disturbances caused by noise and measurement errors in the encoders, the operation of the control is evident, as the vehicle does not slide down because the angular speeds of the wheels are following their references.

**Figure 11:**
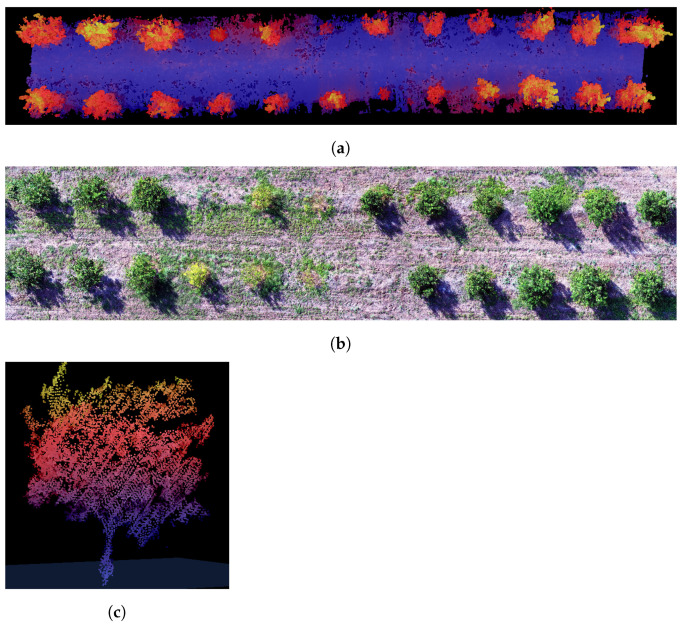
Obtained point cloud from the citrus crop segment: (**a**) Top view of the point cloud, (**b**) RGB image acquired with an aerial vehicle to compare, and (**c**) frontal view of a segmented tree.

After postprocessing, a point cloud was obtained with the 3D description of the route environment; an example of the generated point cloud of the citrus crop with a 20 m wide cut-out is presented in Figure 11. In addition to the geometric information, some additional information layers were acquired such as intensity, a digital representation for every point of the return strength of the laser pulse that generated the point which it is based, in part, on the reflectivity of the object struck by the laser pulse; other obtained information layers were the LiDAR horizontal angle, the LiDAR range, and the number of returns per pulse. Moreover, to better explain the scene, a fragment of an aerial photograph of the same section of the UGV’s route is presented. The generated point cloud consists of 3,405,554 points, of which 3,284,177 points correspond to first echoes, 4478 points correspond to middle echoes and 116,899 points correspond to last echoes. The horizontal angle shows that major objects are illuminated with higher angles because of tilt inclination of the laser. In contrast, the central light beams have a shorter path and mainly illuminate low sections such as the floor. In the same way, with respect to the returns, the beams that illuminated low vegetation and tree areas had the highest number of returns in comparison with solid areas such as the ground.

### 3.2. Crop Parameters Estimation

**Figure 12:**
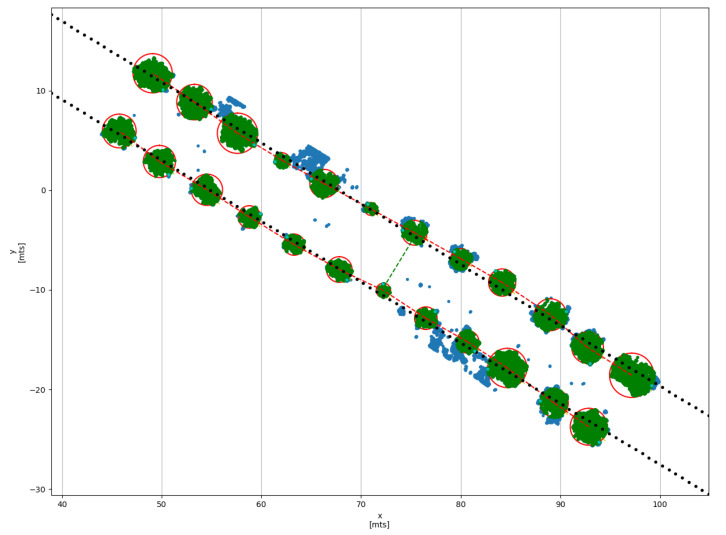
Assisted measurement of crop parameters from the top view of the point cloud with a ground removing in the interactive Python script.

The results of the algorithm implemented on Python described in Section 2.5 are presented in Table 5. The estimation of Crop parameters begins with the uploading of the point cloud generated by the robot to the software. Next, the user selects the $z_{ref}$ parameter in the graphical interface to remove the ground of point cloud and plot an understandable top view (view blue data in Figure 12). Once the scene is displayed, the user uses the mouse cursor to click in the center of each tree where a red “x” symbol appears, The user defines a circle which describes the canopy radius $r_{(i,j)t}$ and forms a cylinder from the crop topmost to the $z_{ref}$ point. When the click is released, a yellow “x” symbol appears and a red circle delimits the cylindrical region $R_{(i,j)t}$. When all trees in current furrow have been selected this step is repeated for a new furrow, by pressing the “space” key on the keyboard to start with a new furrow and continue marking each tree. Once all the trees in all the furrows have been marked the software is able to calculate the crop parameters. The results of this process are presented in the Figure 12.

Finally, the following crop parameters are registered and displayed:

- Canopy height. Measure the height of the canopy and color each point within the region $R_{(i,j)t}$ in green color.
- Crown diameter. From the maximum radius defined graphically by the user with the red rings, the system registers the diameter of the canopy in the corresponding tree.
- Distance between trees. Measure the tree to tree distance into the same furrow and drown a dashed red line between centers of trees $Pt_{(i,j)\left(x,y\right)}$
- Distance between furrows. Measure the furrow to furrow based on the estimation of a first-degree polynomial function in each furrow $S_{F}$, which is drown with dotted black line. The achieved average distance between both straight lines in the evaluated range is illustrated by dashed green line.

The validation for the obtained results was carried out by a comparison of estimated data from our software with respect to the field measurements with a reference photogrammetry method. That information is presented in Table 5. The error of singular parameters as high or diameter of canopy was calculated using the *RMSE* (Root Mean Squared Error) and *R* squared $R^{2}$ as shown in the Equations (42) and (43). For common parameters such as distance between trees or distance between furrows, the comparison was carried out by the experimental error (*Ee*) calculation as the average of measurements with respect to the average of reference measurements, as shown in Equation (44). Additionally, for singular parameters the average value of error between real and estimated information by tree was calculated. The sample data used in this experiment consisted of a group of 24 trees of the citrus family, which are distributed in two rows.

(42) $$RMSE\left(x,\hat{x}\right)=\sqrt{\frac{1}{n}\overset{n}{\underset{i=1}{\sum }}{\left(x_{i}-{\hat{x}}_{i}\right)}^{2}}$$

(43) $$R^{2}\left(x,\hat{x}\right)=\frac{cov(x,\hat{x})}{var\left(x\right)var\left(\hat{x}\right)}$$

where $x_{i}$ and ${\hat{x}}_{i}$, are the field measurement take as reference and the estimated data by software, respectively.

(44) $$Ee\left({\bar{x}}_{e},{\bar{x}}_{r}\right)=\frac{\left|{\bar{x}}_{e}-{\bar{x}}_{r}\right|}{{\bar{x}}_{r}}$$

where ${\bar{x}}_{e}$ and ${\bar{x}}_{r}$ are the average of estimated and real values, respectively.

## 4. Discussion

Geometric characterization of crops provides information about orchard variability and vigor, enabling the farmer to make faster and better decisions in tasks such as irrigation, fertilization, pruning, among others. Appropriate field management requires methods of measuring plant height with a good precision, accuracy, and resolution. With low-cost systems for 3D orchard and small crops mapping based on the combined use of LiDAR devices and terrestrial mobile platforms, we were able to generate 3D models and compute morphological parameters at individual plant level from a cultivated citrus crop. While most of the mapping systems consulted make use of freight vehicles such as tractors, the use of small mobile platforms facilitates mobility between small crop areas and reduces costs. The development of the mobile platform of this work was realized with about 1000 USD. The electronics related to laser mapping, navigation, processing, and powering have varied costs depending on characteristics such as precision, accuracy, or robustness. The LiDAR sensor for outdoor applications has about half of the value in the incidence on the total cost and the navigation unit ~30%, the most influential elements according to the authors of [30], who report the development of a low-cost system for 3d mapping in aerial platforms with a hypothetical selling price about 22,500 USD. In most of the cases of the related works, LiDAR involves investments in excess of 7000 USD and the navigation device in excess of 3000 USD, without taking into account the costs of processing software, computer unit, and battery. However, the proliferation of new embedded systems and low cost devices has facilitated access to these technologies, in our system the total related to navigation, processing and laser registration was around 6500 USD. In addition, the software elements used in the development of this work for both reconstruction and processing are free to use.

This work is an alternative low-cost way of mapping orchards and small crops by generating a 3D model from simple 2D laser devices compared to multi-laser scanners. This reconstruction technology is easily employable in any type of crop where a terrestrial mobile platform can transit, with plants of any size and without the need to include additional infrastructure or intervene in the scanning environments as [31,32] given that it is a non-invasive system. The combined use of the laser measurement system with robotic mobile platforms avoids the need to use robust platforms such as tractors or scooters [14,16,17,33] which cannot always be moved between crops due to the size of the furrows and the type of crop. In addition, the use of the ROS robotics environment makes it compatible with different data computation tools and, as presented in the Section 3.2, it is possible to process the bag of data to generate a 3D model and process it. In our platform, we propose a user-friendly processing tool that reduces human intervention and at the same time determines the individual calculation of morphological parameters in each plant and furrow of the map in which the user is interested. Generally, the data acquisition with the laser scanner in the field worked very well. Nevertheless, problems occur from noise in the point clouds, due to wind, rain, insects, or small particles in the air, reflections on water, and other effects. These issues for terrestrial laser scanning applications in agriculture are also reported in [34]. A resulting limitation of our system due to the use of low-cost mapping and location systems is that the error in the position estimation increases with longer distances, and therefore the quality of the maps is affected in its accuracy, which is why in its current configuration it is only reliable in orchards or small crops. The presented system was able to estimate canopy and crop geometrical parameters at the same time with acceptable correlation coefficients of *RMSE* = 30.8 cm, $R^{2}$ = 0.73 and *Ee* = 9.28%, and *RMSE* = 45.7 cm, $R^{2}$ = 0.64 and *Ee* = 17.29% for the canopy height and canopy diameter, respectively. Knowing those parameters on the tree structure, especially the canopy height and diameter, could be valuable for the planning and optimization of harvesting strategies [35]. For example, depending on the amount of fruits in the top parts of the trees and considering the extra costs involved to pick them (use of ladders or elevation platforms), the farmer could decide not harvest the highest areas. Geometric parameters estimated from raw data were mainly affected by the uncertainties in the laser scanner, the presence of weed plants between trees, the calibration of the setup, and the used pose estimation system. Different works have analyzed the error propagation in scanner laser systems. Similar results were found by the authors of [36] who used an UAV-LiDAR to estimate fresh biomass and crop height for three different crops (potato, sugar beet, and winter wheat). The height estimates worked well for sugar beet ($R^{2}$ = 0.70, *RMSE* = 7.4 cm) and wheat ($R^{2}$ = 0.78, *RMSE* = 3.4 cm). However, for potato plant height ($R^{2}$ = 0.50, *RMSE* = 12 cm), it proved to be less reliable due to the complex canopy structure and the ridges on which potatoes are grown. Small differences on results were found in [8] with an $R^{2}$ of 0.87%, 0.88%, and 0.77% for height, stem diameter, and canopy volume, respectively, the authors of which used a 2D light detection and range (LiDAR) laser scanner system mounted on a tractor and tested on a box with known dimensions for an apple orchard to obtain the 3D structural parameters of the trees, using a real-time kinematic global navigation satellite system (RTK-GNSS). The authors of [14] developed a system which was able to detect and localize more than 80% of the visible fruits, predict the yield with a root mean square error lower than 6% and characterize canopy height, width, cross-sectional area, and leaf area. The system used in their study was based on the Mobile Terrestrial Laser Scanner MTLS described in [37] and replacing the 2D LiDAR sensor with the 3D Velodyne VLP-16. Obtaining an accurate characterization of trees by non-destructive methods at different growth stages provides valuable information that can be used for enhancing precision in orchard management [38,39]. Among the geometrical parameters of the plants, crown diameter and crown height are of particular interest, as both combine the width, height, geometrical shape, and structure of the trees [40]. Such parameters are commonly used for farmers, e.g., when establishing a biomass model for plants [41], in herbicide management, and in pruning directives [42]. However, in this study, the prototype for the data collection was developed using low-cost components, making it accessible. Consequently, laser scanning methods are a promising tool for precision agriculture. The vehicle uses a rocker-bogie suspension and a speed control to facilitate its manipulation and operation by the user avoiding the effects of sudden movements. This system was able to detect geometric variables in citrus trees that can be used in agricultural applications to measure tree growth for individual tree orchard management, while considering mechanical pruning, irrigation, and spraying.

## 5. Conclusions

A system for orchard mapping integrating an UGV and a LiDAR 2D was developed, allowing generating 3D reconstructions of a crop segment and the subsequent determination of spatial characteristics under field conditions such as distance between furrows, distances between trees, tree heights, canopy heights, and canopy diameter, by using an assisted algorithm. The prototype for the data collection was developed using low cost components, making it accessible. The vehicle uses a rocker-bogie suspension and a speed control to facilitate its manipulation and operation by the user avoiding the effects of sudden movements. The reliability of the system was achieved by measuring the variables simultaneously from a generated point cloud with PIX4D and a quadrotor comparing the results with error indicators. For the particular measurements the *RMSE* indicator was determined, obtaining error values of 30.8 cm and 45.7 cm for the height of the trees and the diameter of the crowns, respectively. Additionally the experimental Ee error was determined for the other variables obtaining 0.57% and 6.44% for the tree spacing and furrow to furrow distance, respectively. Future work includes the use of visual and LiDAR SLAM methods to improve pose estimation and consequently the quality of generated point clouds. Furthermore, the development of machine learning techniques to simplify and speed up the calculation of morphological indices in large-scale crops, that can offer a timely use in the agricultural processes of fruit orchards.

## Figures and Tables

**Table 1:** MTLS.

Application	Crop	Sensing Device	Localization Device	Platform	Ref.
Fruit detection, yield prediction, and canopy characterization	Apple	3D Velodyne VLP-16	GPS1200+ Leica RTK-GNSS	MTLS	[14]
Canopy mapping.	Apple	3D Velodyne VLP-16	GPS 3D Robotics	Tractor	[16]
Panicle measurement.	Sorghum	3D FARO Focus X330	RTK GNSS	Tractor	[17]
Precision fruticulture and horticulture.	Vineyards	3D MS Kinect V2	RTK GNSS	UGV	[20]
Fast phenotyping.	Vineyards	2D SICK LMS-400	GPS/IMU - Advanced Navigation, RTK	UGV	[19]
High-throughput phenotyping	Maize	3D Velodyne HDL64-S3	GNSS receiver with two antennas	UGV	[5]
	Ryegrass	2D SICK LMS-400	Here+ V2 RTK GPS	UGV	[18]

**Table 2:** Related parameters to design criteria.

Variables	Values	Unities
$\zeta_{s}$	$\frac{\pi }{6}$	rad
$m_{r}$	20	Kg
$m_{L}$	5	Kg
$v_{min}$	0.7	$\frac{m}{s}$
$a_{min}$	0.5	$\frac{m}{s^{2}}$
μ	0.7	-
*SF*	70	%
*g*	9.8	$\frac{m}{s^{2}}$

**Table 3:** Power consumption of control elements and sensors.

Elements	Power [W]
Computer (Jetson TK1)	3
USB hub TP-Link AC750 wireless router	12
LiDAR UTM-30LX-EW	8.5
Switch TP-Link	4
Servo motor Dynamixel AX-12A	13.5

**Table 4:** Design parameters for wheel speed controllers.

Parameter	Value	Units
*k*	0.1667	rad/s
$\omega_{o}$	5.003	rad/s
ζ	0.9033	-
$T_{s}$	0.1	s
Robustness	>0.8	-
Settling time	<2	s
Overshoot	<0.5	-
$K_{p}$	6.677	rad/s
$K_{i}$	28.656	rad/s
$K_{d}$	0.0734	rad/s

**Table 5:** Experimental error of obtained parameters from point cloud with respect to reference measurements.

Parameter	Reference [m]	Obtained [m]	*RMSE* [m]	$R^{2}$	*Ee* [%]
$h_{c}$	-	-	0.308	0.732	9.282
$d_{c}$	-	-	0.457	0.637	17.294
${\bar{D}}_{t2t}$	5.087	5.116	-	-	0.575
${\bar{D}}_{f2f}$	8.294	7.759	-	-	6.443

## Data Availability

Our code implementation is available online at https://github.com/HaroldMurcia/miniRover_LiDAR_citrush_crop.git, accessed on 25 November 2021.
